# Resilience of breadfruit agro-ecosystems in Hawaiʻi during the COVID-19 pandemic

**DOI:** 10.1186/s43170-022-00125-3

**Published:** 2022-09-04

**Authors:** E. H. Berning, C. V. H. Andersen, O. Mertz, N. Dickinson, M. Opgenorth, N. K. Lincoln, J. H. Rashford, N. Rønsted

**Affiliations:** 1 Department of Geosciences and Natural Resource Management, University of Copenhagen, Copenhagen, Denmark; 2 National Tropical Botanical Garden, Kalāheo, Kauai, HI USA; 3 Natural History Museum of Denmark, University of Copenhagen, Copenhagen, Denmark; 4 National Tropical Botanical Garden, Hāna, Maui, HI USA; 5 Department of Tropical Plant and Soil Sciences, University of Hawai`I at Mānoa, Honolulu, HI USA; 6 College of Charleston, Charleston, SC USA

**Keywords:** Agro-ecosystems, Agroforestry, Breadfruit, Crop diversity, Ecological resilience, Food security, Hawaii

## Abstract

**Background:**

The COVID-19 pandemic is interrupting domestic and global food supply chains resulting in reduced access to healthy diverse diets. Hawaiʻi has been described as a model social-ecological system and it has been suggested that indigenous agro-ecosystems have the potential to be highly productive and resilient under changing land-use and climate change disturbance. However, little research has yet been conducted exploring the disruption and resilience of agro-ecosystems in Hawaiʻi caused by the COVID-19 pandemic. The breadfruit tree (*Artocarpus altilis*; Moraceae) is a signature, multi-purpose-tree of the complex perennial agro-ecosystems systems in Oceania.

**Methods:**

This case study explores the ways in which the breadfruit agro-ecosystems of Hawaiʻi have shown resilience during the COVID-19 pandemic.

**Results:**

Our study suggests that breadfruit has increased its value as a subsistence crop during the COVID-19 pandemic, even in a developed economy like Hawaiʻi, and that resilience of Hawaiian breadfruit agroe-cosystems during a crisis can be supported through cooperatives and food-hubs.

**Supplementary Information:**

The online version contains supplementary material available at 10.1186/s43170-022-00125-3.

## Background

The COVID-19 pandemic is interrupting domestic and global food supply chains resulting in reduced access to healthy diverse diets (WHO 2020; Worstell 2020). While adaptive theory suggests that social-ecological systems are continuously adapting, ecological resilience refers to the degree of disturbance a system can buffer before collapsing and entering a reorganization phase (Cabell and Oelofse 2012; Holling 1973; Worstell and Green 2017). Hawaiʻi has been described as a model social-ecological system (Kirch 2007) and it has been suggested that indigenous agro-ecosystems have the potential to be highly productive and resilient under changing land-use and climate change disturbance (Kurashima et al. 2019). However, little research has yet been conducted exploring the disruption and resilience of agro-ecosystems in Hawaiʻi caused by the COVID-19 pandemic (Fardkhales and Lincoln 2021; Lee and Milne 2020; Miles and Merrigan 2020; Worstell and Green 2017).

The state of Hawaiʻi has one of the highest poverty rates in the United States of America at 13.4% (US Census Bureau 2020), which contributes to an array of food consumption problems for low-income and indigenous communities including moderate to high rates of household food insecurity and frequent consumption of ultra-processed, low-quality fast foods leading to health issues (Ho-Lastimosa et al. 2020; Miles and Merrigan 2020; US Census Bureau 2020).

Hawaiʻi’s local food system is being tested by the crisis caused by COVID-19 which in March 2020 imposed stay-at-home orders, strict travel restrictions, and business closures, lasting more than a year (Fardkhales and Lincoln 2021). According to the Hawaiʻi Department of Agriculture, estimates in early summer of 2020 showed that local farmers and ranchers have seen a 50% decline in sales (Lee and Milne 2020). For small farms in Hawaiʻi the shutdown of physical sales channels such as farmers markets has been especially problematic while the dependence on sales channels such as hotels, restaurants and schools has been the main challenge for medium to large sized farms. It has forced farms to change their strategy in order to respond to the changing consumer demand (Fardkhales and Lincoln 2021). Hawaiʻi only produces about 10% of its food locally (Needham and Lincoln 2019) and it is an explicit goal defined by The Hawaiʻi Green Growth Local2030 Hub, a public–private partnership committed to advancing economic, social and environmental goals, to double the local food production by 2030 (Hawaiʻi Green Growth 2018).

**Fig. 1:**
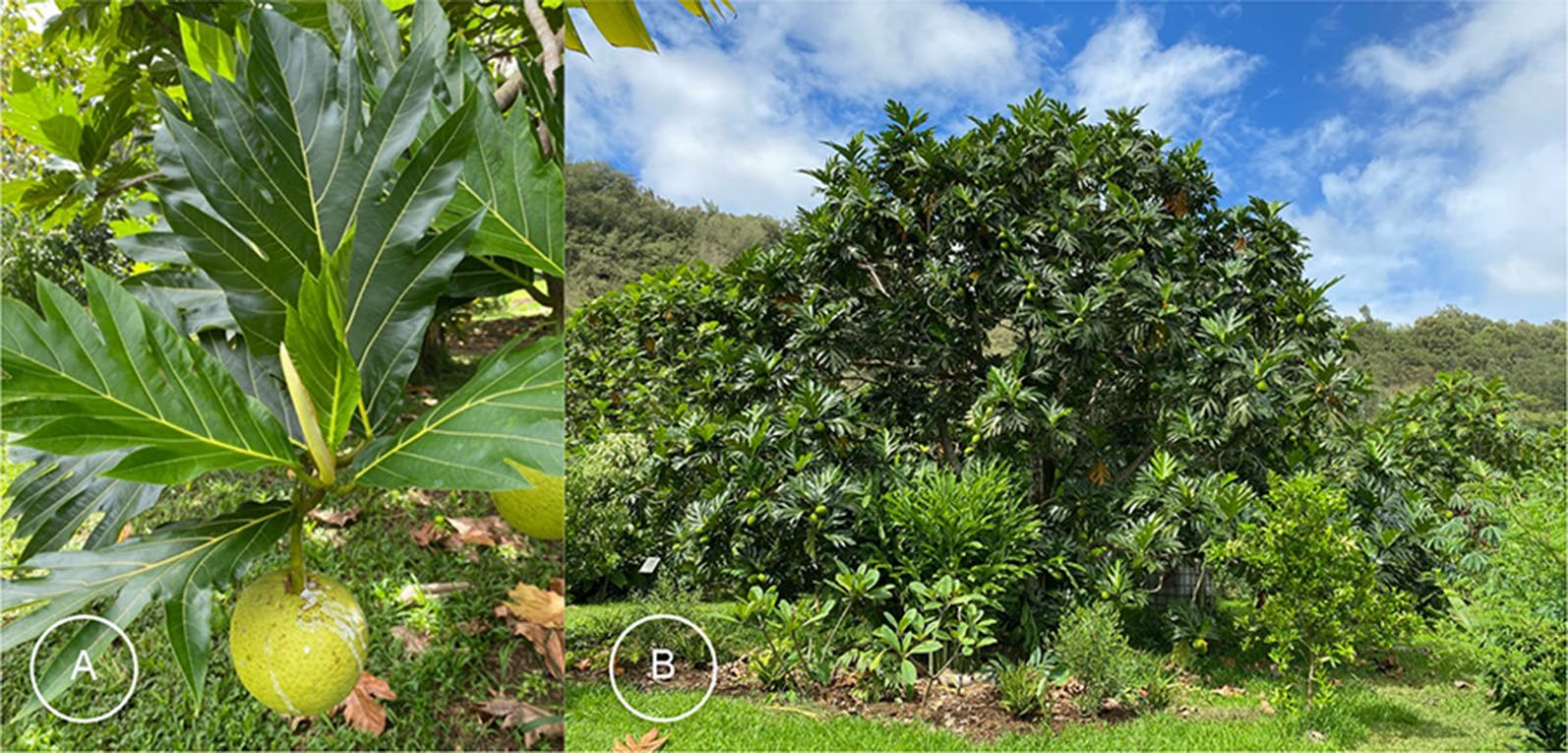
A. Breadfruit, *Artocarpus altilis*. B. The Regenerative Organic Breadfruit Agroforest (ROBA) or agro-ecosystem demonstration plot in the National Tropical Botanical Garden, Kauaʻi, Hawaiʻi, is composed of more than 100 species. Photos by the Breadfruit Institute, National Tropical Botanical Garden

The sudden disruption caused by the COVID-19 pandemic, emerging in early 2020, has brought to light the importance of Hawaiʻi’s breadfruit agro-ecosystems (Fig. 1). Breadfruit (*Artocarpus altilis* (Parkinson ex F.A.Zorn) Fosberg; Moraceae; ‘Ulu in Hawaiian; Fig. 1) is a Polynesian staple food and is seen as an underutilized, nutritious, low-maintenance and productive tree crop which has the potential to contribute to greater food security in Hawaiʻi as well as being a solution to agricultural sustainability issues in the state (Jones et al. 2011; Liu et al. 2015; Needham et al. 2020; Needham and Lincoln 2019; NTBG 2019; Ragone et al. 2016).

Breadfruit produces abundant, nutritious fruit, which are high in carbohydrates and a good source of fiber, vitamins, minerals, and protein (Liu et al. 2015; Ragone 2011). The fruit is typically cooked and consumed as a starchy staple when firm and mature. Fruit quickly ripens in a few days after harvest with limited shell-life. Covering fruit with ice or water can delay ripening for several days to more than a week. Ripe fruit can be eaten raw or cooked, fermented, processed into chips and other snacks, dried into flour or starch, and minimally processed or frozen. Breadfruit flour can be partially substituted for wheat flour in many bread, pastry, and snack products. Seeds cooked in the fruit are high in protein, relatively low in fat and a good source of vitamins and minerals (Ragone 2011).

Breadfruit production is seasonal with most varieties producing one or two crops per year. The main crop typically occurs during the hot, rainy, summer months, followed by a smaller crop 3–4 months later (Ragone 2011). Historically, few reports exist of breadfruit pests and diseases, which were traditionally treated by burning the impacted tree (Lincoln et al. 2021). With increased globalization and mobility within breadfruit growing regions, an increasing number of severe diseases have been documented for breadfruit. These diseases attack both leaves, fruits, stems and roots, but can largely be controlled by integrative pest management including soil and water management, pruning and spacing thus limiting the need for chemical control (Lincoln et al. 2021).

The breadfruit tree is a signature, multi-purpose-tree of the complex perennial agro-ecosystems systems in Oceania (Quintus et al. 2019) with several hundred documented cultivars (Ragone and Wiseman 2007). Breadfruit agriculture is found in various forms in Hawaiʻi, including as food forests (Lincoln 2020), as intensively managed diversified orchards (Lincoln and Ladefoged 2014), and as individual trees in home gardens (Meilleur et al 2004). Breadfruit agriculture was considered widespread before European arrival with breadfruit groves reported around the islands and a belt of an estimated 100,000 trees in the Kona region alone (Lincoln and Ladefoged 2014). Following a significant decline in traditional agriculture, breadfruit has seen a dramatic rise growing rapidly from fewer than 500 trees in commercial plantings 25 years ago, to more than 8,000 trees today (Langston and Lincoln, 2018; Lincoln et al. 2021) and more than 120 breadfruit farmers are registered as members of the Hawaiʻi ‘Ulu Cooperative (2022). However, many farmers could be considered backyard growers or having orchards, which is not considered diversified agroforestry (Langston and Lincoln, 2018). The estimated number of breadfruit farmers in Hawaiʻi is maybe about 80–100, but no systematic survey of farms has been conducted for verification.

This paper addresses breadfruit agro-ecosystems in Hawaiʻi managed with the intention of producing, distributing or consuming food (Elevitch et al. 2018; Elevitch and Ragone 2018). There are examples of breadfruit allowing intercropping of more than 120 useful species (Elevitch and Wilkinson 2000), and such agro-ecosystems are being recognized as a holistic food production system such as an agroforest along with environmental, social, and economic benefits (Elevitch et al. 2018; Winter et al. 2020). However, monoculture cultivation of breadfruit emerging approximately a decade ago is expanding, and these systems lack the regenerative characteristics of diverse, multistory breadfruit agro-ecosystems (Elevitch et al. 2018). Therefore, it is relevant to examine the benefits of breadfruit agro-ecosystems perceived by practitioners, especially during the COVID-19 crisis.

As outlined above, there has been limited research on the role of breadfruit and other staple crops in ensuring resilience of local communities during pandemics. This case study explores the ways in which the breadfruit agro-ecosystems of Hawaiʻi have shown resilience during the COVID-19 pandemic.

## Methods

The case study was conducted as an online multiple-choice questionnaire survey (Additional file 1: Table S1). In-person interviews with farmers were not possible due to the travel and social interaction restrictions presented by COVID-19. The location of the case study was the state of Hawaiʻi in the USA and the survey was conducted throughout November 2020.

The questionnaire consisted of 58 questions (Additional file 1: Table S1). The initiating questions in the survey were used to establish the background of each respondent. The more familiar term agroforest was generally used in place of agro-ecosystems throughout the questionnaire to the farmers. Following this, a series of questions establishes the current farming practices of each farm as well as the social component of the farm management e.g., sharing of equipment, number of sellable products and organization in networks. Several questions in the survey revolved around the issue of COVID-19 and the impact that the pandemic has had on breadfruit farm operations in Hawaiʻi.

Because agro-ecosystems embody all the complexity that a socio-economic system can have, it is impossible to account for all the factors contributing to resilience in these systems and therefore Darnhofer et al. (2010) argues that surrogates and indicators can only be used to assess resilience rather than to measure it. Building on this insight, Cabell and Oelofse (2012), suggest an index of behavior-based indicators compiled from characteristics of resilient socio-economic systems in literature that is concerned with socio-economic resilience in agro-ecosystems in different contexts (Additional file 1: Table S3). In order to depict how circumstances are affecting breadfruit farms, questions were designed based on Cabell and Oelofse’s (2012) agroecosystem resilience framework to let farmers consider whether certain “behaviors” stressed by the indicator framework had an effect on the robustness of their farm operation. A behavior of an agroecosystem farmer is here analogous to an agricultural practice used by Cabell and Oelofse (2012) as indicator of an abstract concept of resilience as detailed in Additional file 1: Table S3.

Other questions were designed to give an indication of new or increased practices arising during the COVID-19 pandemic related to agroecosystem resilience.

The survey questionnaire was created using SurveyXact by Ramboll software made available by the University of Copenhagen. We initially conducted two pretests of the survey in order to mitigate any ambiguity in the survey questions. The distribution of the questionnaire to breadfruit farmers was done through a form of snow-balling sampling technique through key stakeholders in the breadfruit growers’ network in Hawaiʻi including the Hawaiʻi Tropical Fruit Growers, the Breadfruit Institute of National Tropical Botanical Garden, and the Hawai’i ‘Ulu Cooperative, as well as to farmers in the author's own network. A hyperlink allowed for participants to re-distribute the survey. The definition of a breadfruit farmer is not well defined in terms of number of trees or agricultural practices used. Langston and Lincoln (2018) considered a breadfruit “farmer” to have at least 6 trees. In this study, we allowed self-identification as a farmer to be the inclusion criterion. Initial questions in the survey sought to make sure all participants were breadfruit farmers, situated in Hawaiʻi.

The survey was distributed to a total of 66 farmers or about 66–80% of the estimated number of breadfruit farmers in Hawaiʻi. Twenty-nine participants corresponding to 29–36% of the estimated number of breadfruit farmers in Hawaiʻi answered some questions, 14 participants (14–18% of the estimated number of farmers in Hawaiʻi) answered all questions, and 23 people received the survey but did not answer any questions. The participants answering the location question were geographically distributed on the Hawaiian Islands with 9 from the Island of Hawaiʻi, 3 from Molokaʻi, 4 from Kauaʻi, 2 from Maui and 2 from Oʻahu. While the sample size of survey questions is limited and irregular (ranging from n = 14 to n = 29), they are sufficient to outline some broad trends (Additional file 1: Table S2).

## Results and discussion

### Farmers’ perception of the role of breadfruit during COVID-19 and the future of the crop in Hawaiʻi

The subsistence value of breadfruit has increased during COVID-19 according to 13 of 14 participants (93%), and this result resonated with previous conclusions of breadfruit being an important subsistence crop (Needham and Lincoln 2019; Ragone 2018).

**Fig. 2:**
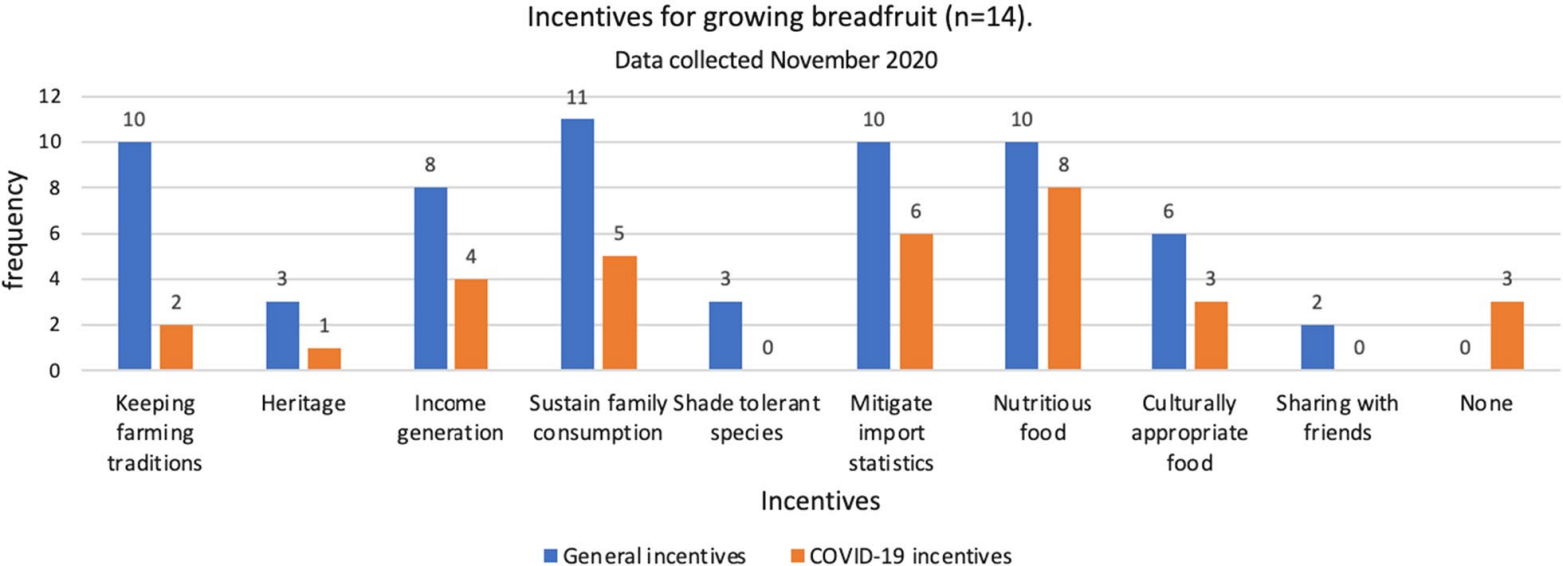
Incentives for growing breadfruit in general and newly emerged incentives as an effect of the COVID-19 pandemic

However, the finding that breadfruit gains importance as a subsistence crop during a crisis such as the COVID-19 pandemic is novel, and asserts the importance and potential of breadfruit as a nutritious, high yielding crop which can mitigate food security issues (Ragone et al. 2016), even in a developed economy like Hawaiʻi. In addition, we found that the incentive of growing breadfruit to sustain family consumption needs has been reinforced as an effect of the pandemic (Fig. 2).

Breadfruit has gained importance for local communities during COVID-19. The desire of customers to buy local produce as a direct reaction to the COVID-19 pandemic has increased according to 10 of 14 participants (71%). Though this question was not specifically directed at the demand for breadfruit, it indicates that the interest for local farm produce has increased during the pandemic. Furthermore, 3 of 14 participants (21%) responded that they would increase the amount of breadfruit trees on their farm as an effect of the COVID-19 pandemic.

This trend is supported by findings of Fardkhales and Lincoln (2021) who conclude that 83% of food hubs increased their purchasing of local food from Hawaiian farmers during COVID-19. In effect the COVID-19 pandemic has had a positive impact on the narrative around local food which could offset important change for the future of the breadfruit crop in Hawaiʻi thereby increasing consumer acceptability of breadfruit in line with needs expressed in a study by Lysák et al. (2019).

Additionally, the circumstances have shed light on the vital role that breadfruit can play in a food emergency crisis, for example replacing rice as a staple crop at Hawaiian food banks, when there was a rice shortage in the US mainland (Fardkhales and Lincoln 2021). Our results show that a majority of participants express that being embedded in the community benefits the farm operation during the COVID-19 pandemic. Milestad and Darnhofer (2003) argue that farms with local networks that are rooted in the local community can build a strong relationship with consumers and thereby provide options for direct marketing.

Three of 14 participants (21%) responded that they would increase the amount of breadfruit trees on their farm as an effect of the COVID-19 pandemic. Although this is a relatively low number of respondents, the result is worth paying attention to, as it indicates at least that some farmers predict an increased production need as an effect of the crisis.

Based on the limited number of respondents (n = 19), farmers involved in a cooperative (6 farmers) utilize 87% and sell 52% of their harvested breadfruit on average, while farmers not involved with a cooperative (n = 13) only utilize 64% and sell 28% of their harvested breadfruit.

**Fig. 3:**
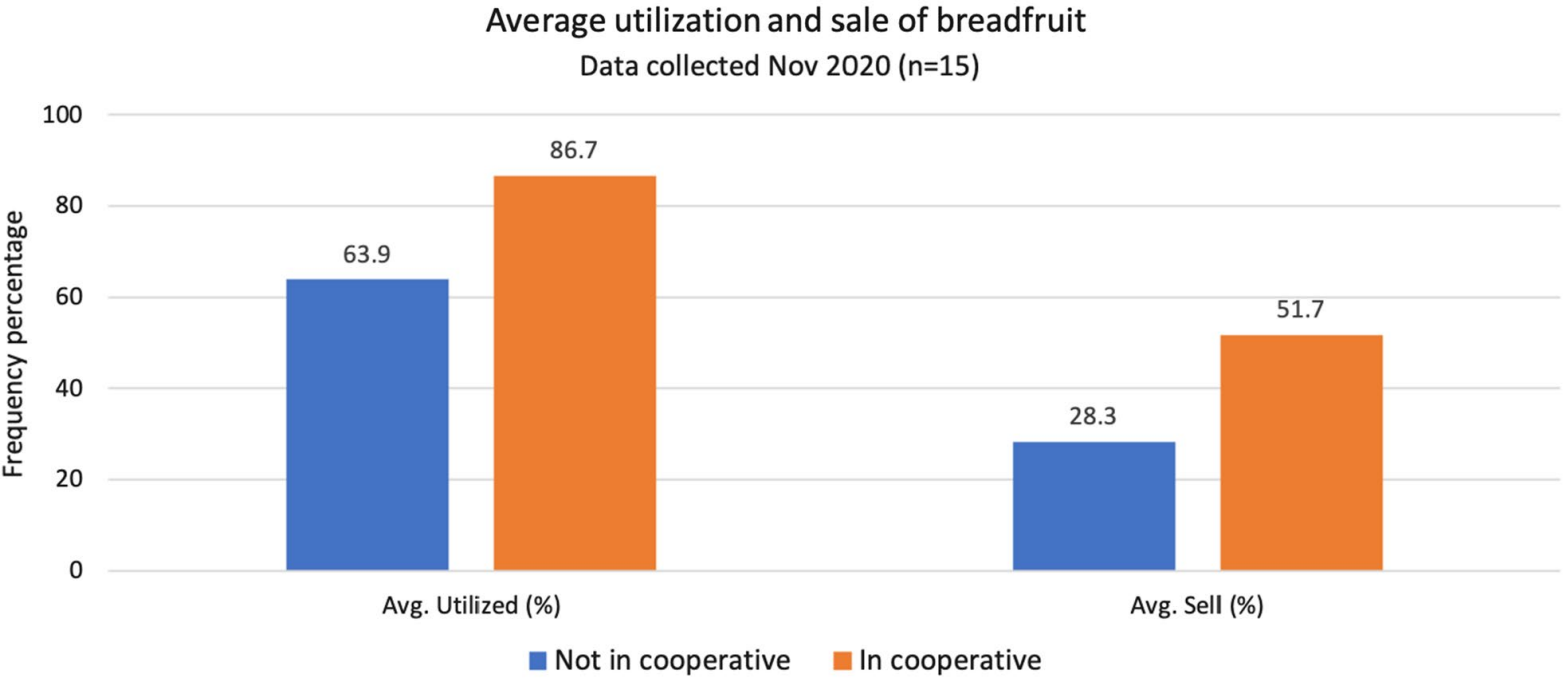
Diagram showing the difference in average utilization of breadfruit and average amount of sold breadfruits in the total harvest, for participants in cooperatives and participants not in cooperatives. Utilization includes selling, private consumption, donating, and feeding animals as opposed to waste

The waste percentage of the entire sample combined is 27% (Fig. 3). Whereas all types of fruit production generally have a waste proportion, the relatively low utilization percentage outside the cooperatives especially is in line with findings by Ragone et al. (2016) suggesting an unused capacity of breadfruit. While we did not investigate reasons for waste, we speculate that many new trees have been planted but are not yet productive, whereas many older trees have a size that exceeds harvestability, some fruit is lost to disease/fungal pressure, and some fruit harvested is not a high enough grade for use in sale or distribution (Langston and Lincoln 2018). Additional reasons for farmers not harvesting everything is possibly competing demands on time and lack of capacity. From the cooperative perspective the demand by far outstrips the supply at the moment (Hawaiʻi ʻUlu Cooperative, 2022). For those not in a cooperative access to markets may be the greatest reason it is not all harvested, which emphasizes the importance of the cooperatives and other solutions to increase market access.

### Profitability of outlets for breadfruit produce

**Fig. 4:**
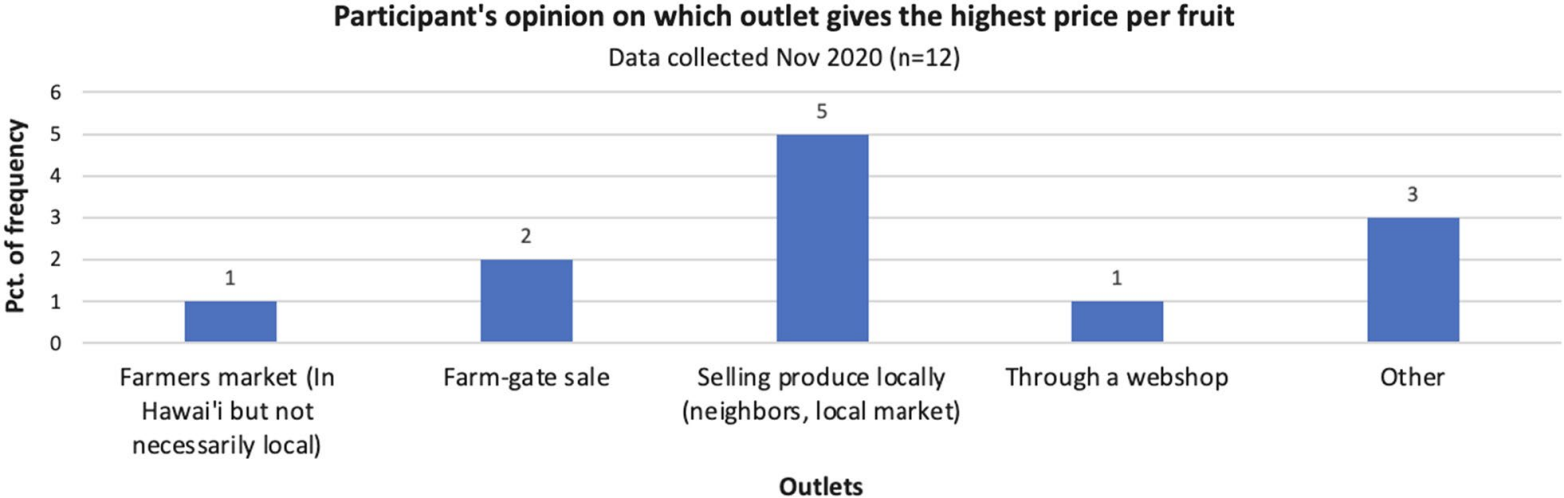
Distribution of participants’ opinion on which outlets give the highest price per breadfruit. Only participants who sell their breadfruit were asked to answer the question

To gain knowledge about how the various outputs of breadfruit vary in profitability from the farmers perspective, we asked participants which outlet gives them the highest price per breadfruit. The responses indicated that selling produce locally gives the highest price per breadfruit (42%) (Fig. 4), which might explain why strong ties with the community, in which the farm exists, provides a connection which can serve as an important direct sales outlet, during the COVID-19 pandemic and beyond. It is important to bear in mind that half of the participants in the survey are not producing breadfruit commercially but merely for subsistence reasons, which means that selling produce to locals and neighbors is potentially the only available outlet for half of the participants.

Fardkhales and Lincoln (2021) found that the Hawaiʻi ʻUlu Cooperative had an increase in total sales by 25% after the outbreak of the COVID-19 pandemic. The cooperative also had an increase in suppliers from 80 before March 2020 to 100 after June 2020. The sales in the cooperative went from being mainly reliant on institutions and the service industry to community supported agriculture (CSA’s), defined by the US Department of Agriculture as a community of individuals who pledge support to a farm operation so that the farmland becomes, either legally or spiritually, the community’s farm, with the growers and consumers providing mutual support and sharing the risks and benefits of food production (Woods et al., 2017), as well as collaborations with other food hubs and food banks (encompassed in the category food distributors, thereby making most of their outlets direct-to consumer oriented).

### Benefits of agro-ecosystems for community resilience

**Fig. 5:**
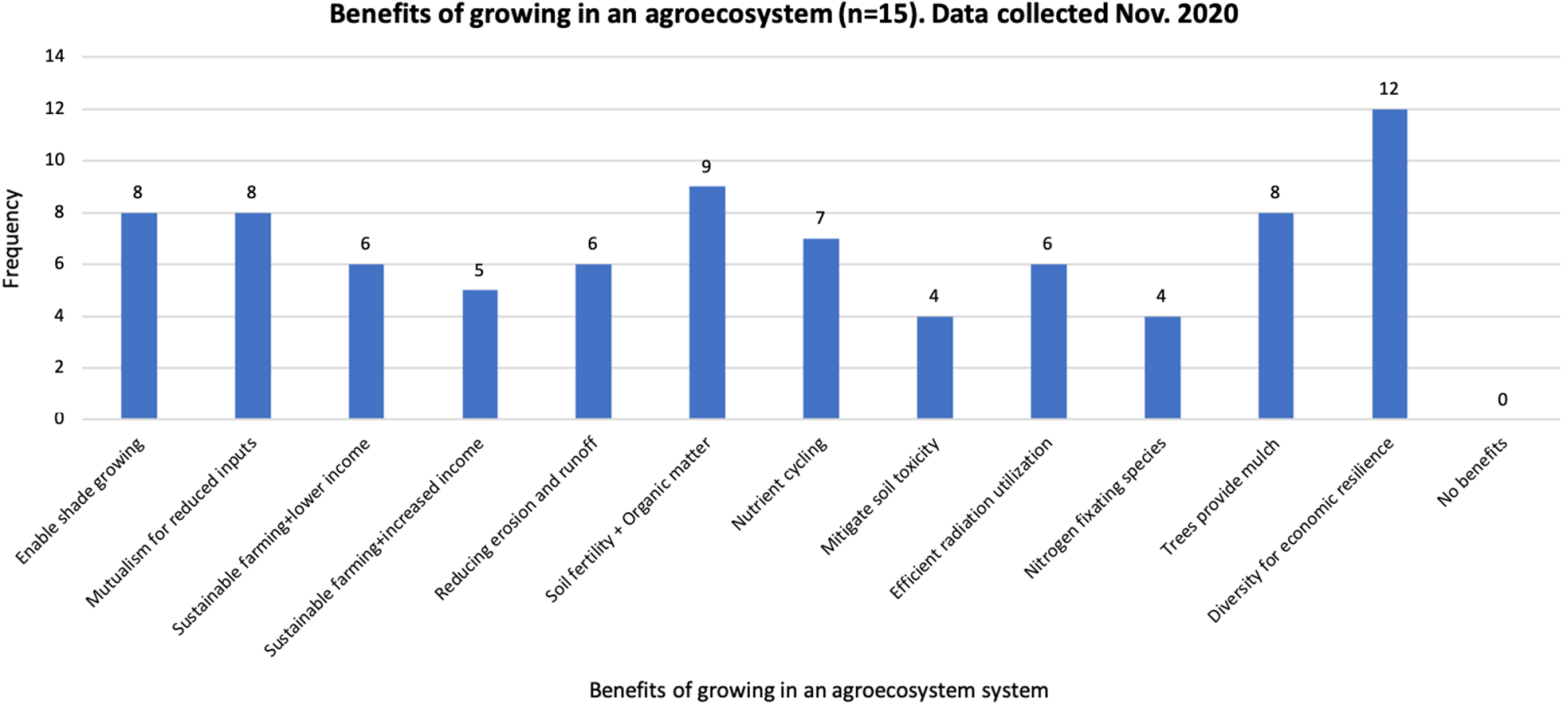
Benefits of growing breadfruit in an agro-ecosystem (agroforestry system), with frequency on the y-axis and benefit-categories on the x-axis (n = 15)

Ten of 15 participants (67%) answered that they employ some configuration of agro-ecosystems, as opposed to monoculture or non-integrated diverse farming, and 7 of 10 (70%) participants said that their agro-ecosystems practice has benefitted their farm operation during COVID-19. Participants identified 12 socio-economic and environmental benefits of growing breadfruit in agro-ecosystems with “Diverse farming for economic resilience” being the most commonly perceived benefit of agro-ecosystems (12 of 15; 80%) (Fig. 5).

The number of sellable products that surveyed farmers’ agro-ecosystem produce ranged from 1 to  > 10, with 2–4 being most common (40%, n = 10). Among the most important crops of participants' agro-ecosystems were banana, avocado, citrus and cassava (Additional file 1: Table S2). Traditional Hawaiian agro-ecosystems crops like ti, turmeric, kava, sugarcane and taro were also mentioned (Additional file 1: Table S2) (Elevitch and Ragone 2018; Kurashima and Kirch 2011; Lincoln and Ladefoged 2014).

The Maʻafala variety was the most used (17 of 24 participants, 71%) of 13 listed breadfruit varieties reported to be used by the farmers and is known for having a high protein content and quality (Additional file 1: Table S2; Jones et al. 2011). ‘Ulu fiti, which is known for high mineral content, especially iron, was being used by 9 of 24 farmers (38%). The more unseasonal varieties such as Meion, Meinpadakh, and Toneno (Liu et al. 2014) were only reported used by one farmer each, whereas the Pua’a variety was used by 3 of 24 farmers. Farmer’s reported harvesting in all months with August to December being peak season and March–June being low season. This suggests there is a potential for increasing benefits from planting more varieties including unseasonal varieties (Jones et al. 2011). Access to diverse varieties is still limited due to lack of capacity and distribution chains, but more than 150 different varieties are being preserved in ex situ collections, primarily by the Breadfruit Institute in NTBG’s Kahanu Garden in Hana, Maui (Global Crop Diversity Trust, 2007).

## Conclusion

This case study shows that the COVID-19 pandemic provides an opportunity to understand the significance of breadfruit in Hawaiian agro-ecosystems and how the breadfruit contributes to community resilience through its role as an important subsistence crop during times of crisis. Viewing breadfruit farming systems from a community resilience perspective provides the opportunity to investigate whether farmers and farms are endowed with the capacity for transformation and adaptation. Though a systematic resilience assessment of Hawaiian breadfruit farms proved difficult to operationalize due to low response rate, our study revealed a potential for building farm resilience.

Our study suggests that breadfruit has increased its value as a subsistence crop during the COVID-19 pandemic and that resilience of Hawaiian breadfruit agro-ecosystems during a crisis can be supported through cooperatives and food-hubs and by increasing the number of farmers growing non-seasonal varieties or varieties with complimentary seasons, to ensure fruit supply throughout the year, ideally in an ecologically diverse agroecosystem setting. Additional support can involve farmer training programs, cooperative extension services, and accessibility to affordable agricultural land to own or lease in order to justify the multi-year investment planting tree crops requires prior to harvest. Thus, sustaining and nurturing a steady local demand is a way in which resilience can be built. The results provide insights into agroecosystem vulnerabilities and important behaviors and practices increasing resilience of the agroecosystem during a crisis. The findings also underline the importance of incorporating resilience thinking in agro-ecosystems, and the role of breadfruit as an underutilized crop for food security and resilience.

## Supplementary Information

**Additional file 1: Data S1.** Survey questionnaire. **Data S2.** Complete data of all survey questions excluding participants contact information. **Data S3.** Agroforestry characteristics relating to distinct resilience indicators (Cabell and Oelofse 2012).

## Data Availability

The questionnaire with all collected anonymized responses is included as supplementary data online.
